# Comparison of retinal nerve fiber layer thickness in patients having pseudo exfoliation syndrome with healthy adults

**DOI:** 10.12669/pjms.326.11075

**Published:** 2016

**Authors:** Naila Yasmeen, Nauroz Fatima, Qamar-ul-Islam

**Affiliations:** 1 Dr. Naila Yasmeen, FCPS. Armed Forces Institute of Ophthalmology, Rawalpindi, Pakistan; 2 Dr. Nauroz Fatima, FCPS. Combined Military Hospital, Rawalpindi, Pakistan; 3 Dr. Qamar-ul-Islam, FCPS. PNS Shifa Naval Hospital, Karachi, Pakistan

**Keywords:** Pseudo exfoliation syndrome, Intraocular pressure, Optical coherence tomography, Retinal nerve fiber layer thickness

## Abstract

**Objective:**

To compare mean retinal nerve fiber layer (RNFL) thickness in patients having pseudo exfoliation (PXF) with normal age matched controls using spectral domain optical coherence tomography (SD-OCT).

**Methods:**

This was a case control study conducted at Armed Forces Institute of Ophthalmology (AFIO) Rawalpindi from 12 June 2013 to 12 January 2014. Seventy eyes (Group A - 35 patients with PXF and Group B - 35 healthy age matched subjects) of more than 40 years of age were included in the study. Intraocular pressure (IOP) was measured with Goldmann applanation tonometer (GAT) and peripapillary RNFL thickness was measured in four quadrants with SD-OCT (Topcon 3D OCT-1000 Mark II) in all subjects. Data was analyzed using the SPSS version 14.

**Results:**

Mean age of group A (PXF patients) was 65.63 ± 8.47 years and of group B (Healthy subjects) was 64.31 ± 6.51 years (p = 0.470). Both groups were gender matched with male preponderance (p = 0.673). Mean IOP in each group was 13.80 ± 2.59 mm Hg, and 13.49 ± 2.07 mm Hg respectively (p= 0.578). Mean average peripapillary RNFL thickness was 77.46 ± 12.17 µm in group A and 83.96 ± 10.58 µm in group B. Statistically significant differences were detected between two groups for mean average RNFL thickness (p= 0.020) and mean RNFL thickness in inferior quadrant (p=0.014).

**Conclusion:**

PXF patients with normal IOP and visual fields have thin RNFL as compared to healthy age matched controls. Therefore routine assessment and follow up of PXF patients with OCT may help in early diagnosis of PXF glaucoma.

## INTRODUCTION

Open angle glaucoma is known as ‘silent thief of eyesight’ and early diagnosis is of paramount importance in preventing blindness related to this disease. Pseudo exfoliation (PXF) syndrome is one of the commonest cause of secondary open angle glaucoma accounting for 20-25% of all open angle glaucomas worldwide.^1^-^3^ Prevalence of PXF increases markedly with age and has considerable racial variations with reported incidence between 4.48% to 6.45% in Pakistani population.^4^,^5^ Apart from elevated intraocular pressure (IOP), various pressure independent factors represent the main risk factors for loss of retinal nerve fiber layer (RNFL) with subsequent glaucomatous damage in PXF syndrome. These factors include impaired ocular and retro bulbar perfusion, abnormalities of elastic tissues of lamina cribrosa, increased protein concentration of aqueous humour, 1pronounced melanin deposition and exfoliation material itself.^1^-^3^,^6^

In glaucoma structural damage of optic nerve head and RNFL may precede functional loss and it is estimated that between 30-50% of retinal ganglion cells may be lost before detectable changes in visual fields are evident.^7^-^9^ SD-OCT is a rapidly evolving robust technology that provides high resolution, quantitative and reproducible measurements of RNFL and retinal ganglion cell complex having high sensitivity and specificity for differentiating normal eyes from patients with early glaucoma.^7^,^9^ PXF without glaucoma is associated with thin RNFL compared to age matched healthy controls. Sorkhabi et al found significantly thinner average RNFL in PXF group than control (94.36 ± 8.70 µm vs 100.80 ± 6.68 µm) (p= 0.002).^3^ Another study revealed significantly thinner RNFL in PXF group (86.52 ± 19.7 µm) compared to healthy eyes (99.21±20.21 µm) in all quadrants (p < 0.05) except the nasal quadrant (p > 0.1).^2^ Fatima et al found average RNFL thickness being 30% less in glaucomatous eyes as compared to normal eyes in Pakistani population.^10^ However, no local data was found on estimation of RNFL thickness in patients with PXF without glaucoma. The objective of this study was to compare mean RNFL thickness in patients having PXF syndrome with normal age matched controls using SD-OCT.

## METHODS

This case control study was conducted at Armed Forces Institute of Ophthalmology (AFIO) Rawalpindi from 12 June 2013 to 12 January 2014 after approval of hospital ethics committee. Non probability consecutive sampling technique was used and calculated sample size was 31 in each group keeping level of significance as 5, power of test as 90, population mean as 94.36, control mean as 100.8 and population SD as 8.7.^3^ The study was conducted in accordance with the ethical considerations given in Helsinki declaration and written informed consent was obtained before examination. Seventy eyes were divided into two groups of 35 subjects each. In group A subjects of age more than 40 years with PXF and normal IOP (< 21 mm Hg), normal optic disc appearance (cup: disc ratio ≤ 0.3) and normal visual fields were included. Group B included normal subjects of more than 40 years of age with no evidence of ocular pathologies and normal IOP at three different successive measurements. In control group one eye was selected randomly for analysis. Subjects with refractive error > ± 5 diopters, previous intraocular surgery, trauma or laser treatment, media opacities interfering with visualization and OCT image capturing, retinal pathology, family history of glaucoma and history of diabetes mellitus or hypertension were excluded.

Complete history and comprehensive ophthalmic examination was carried out in each subject. IOP was checked with Goldmann applanation tonometer between 0900-1200 hrs to eliminate the effect of diurnal variation. Humphrey (central 30-2) visual field examination was performed in each subject. RNFL thickness was measured with SD- OCT machine (Topcon 3D OCT-1000 Mark II) and circular OCT tomograms were acquired around the optic disc at diameter of 3.4 mm. RNFL thickness was presented on a circular chart with 4 equal 90° hour sectors each representing 1 quadrant displaying RNFL thickness (micrometers) within each sector. All the data was entered on a pre devised data collection proforma.

Statistical analysis of the data was done using SPSS version 14.0. All the data were tested for normality before analysis. Descriptive statistics i.e. means ± standard deviation (SD) for quantitative variables (age, RNFL thickness, IOP) and frequencies and percentages for qualitative variables (gender) were used. Fischer exact test was used for analysis of qualitative variables between groups. Independent sample t-test was applied to compare the mean difference of RNFL thickness between Group A and Group B. A p value of ≤ 0.05 was considered significant.

## RESULTS

**Fig.1:**
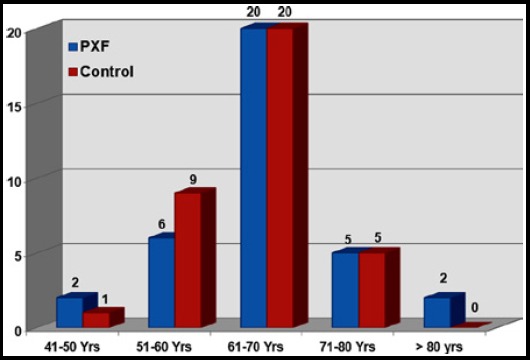
Age Spectrum.

Mean age of study population in group A was 65.63 ± 8.47 years (range: 48 – 85 years) and group B was 64.31± 6.51years (range: 45 – 75 years) respectively (p = 0.470) with majority of patients in both groups were in their 7^th^ decade of life (Fig.1). Both groups showed male preponderance, but were similar in terms of IOP (Table-I). Mean of average peripapillary RNFL thickness was 77.46 ± 12.17 µm (range: 52-103 µm), whereas, mean peripapillary RNFL thickness of superior, inferior, temporal and nasal quadrant was 96.17 ± 18.58 µm (range: 52-146 µm), 88.43 ± 20.36 µm (range: 45 -132 µm), 63.46 ± 10.91 µm (range: 49 - 89 µm) and 61.77 ± 14.34 µm (range: 42 -103 µm) respectively in group A. In group B, mean of average peripapillary RNFL thickness was 83.96 ± 10.58 µm (range: 61-103 µm), whereas, mean peripapillary RNFL thickness of superior, inferior, temporal and nasal quadrant was 103.31 ± 12.36 µm (range: 80-127 µm), 100.46 ± 19.64 µm (range: 55 -128 µm), 68.40 ± 12.36 µm (range: 49 - 95 µm) and 63.66 ± 10.58 µm (range: 40 -103 µm) respectively. Statistically significant differences were detected between two groups for mean average RNFL thickness (p= 0.020) and mean RNFL thickness in inferior quadrant (p=0.014) (Table-II).

**Table-I:** Group wise demographic data (n=70).

Characteristic	PXF (Group A) (n=35)	Control (Group B) (n=35)	p Value
Age (Years) Mean ± SD	65.63 ± 8.47	64.31± 6.51	0.470
Gender (Male/Female)	33 / 2	31 / 4	0.673
IOP (mm Hg) Mean ± SD	13.80 ± 2.59	13.49 ± 2.07	0.578

**Table-II:** Group wise distribution of peripapillary RNFL thickness.

Quadrant	PXF (Group A) (n=35)	95 % CI	Control (Group B) (n=35)	95 % CI	p Value
Average (µm) Mean ± SD	77.46 ± 12.17	73.28 – 81.64	83.96 ± 10.58	80.32 – 87.59	0.020
Superior (µm) Mean ± SD	96.17± 18.58	89.79 – 102.55	103.31 ± 12.36	99.07 – 107.56	0.063
Inferior (µm) Mean ± SD	88.43 ± 20.36	81.43 – 95.42	100.46 ± 19.64	93.71 – 107.20	0.014
Nasal (µm) Mean ± SD	61.77 ± 14.34	56.84 – 66.70	63.66 ± 13.73	58.94 – 68.37	0.576
Temporal (µm) Mean ± SD	63.46 ± 10.91	59.71 – 67.21	68.40 ± 12.36	64.15 – 72.65	0.081

## DISCUSSION

PXF syndrome is a complex systemic disorder of the extracellular matrix primarily affecting the eye and visceral organs characterized by the deposition of fibrillar material on all anterior segment structures. It is one of the commonest causes of secondary open angle glaucoma and in the Blue Mountains Eye study, patients with PEX in either eye have a two to threefold higher risk of open angle glaucoma.^11^,^12^ Although blindness is the most feared outcome, there is growing evidence that even mild visual field loss may have an adverse effect on quality of life. Perimetry which was considered the gold standard for diagnosing glaucoma may not detect visual field defects until substantial numbers of axons are already lost. OCT has a well known role in diagnosing pre perimetric glaucoma. Recently Lisboa et al evaluated the ability of SD OCT to detect pre perimetric glaucoma. The study found that SD OCT was able to discriminate eyes with pre perimetric glaucoma from those with suspected glaucoma.^13^

Prevalence of PXF increases markedly with age.^5^ In our study 94.3% patients in PXF group were males with mean age of 65.63 ± 8.47 years. A male preponderance between 53.3 – 60% with mean ages between 56.2 – 73.9 years was reported in various other studies.^1^,^2^,^6^ Studies on Pakistani population with PXF syndrome also reported male preponderance with ages ranging from 50 - 82 years and mean ages being higher in PXF population as compared to normal controls.^4^,^5^

Our study revealed significantly thinner mean average peripapillary RNFL thickness (77.46 µm vs. 83.96 µm) and mean RNFL thickness in inferior quadrant (88.43 µm vs. 100.46 µm) in patients having PXF with normal IOP as compared with age matched healthy adults using SD OCT. Yuksel et al compared patients in three groups i.e. unilateral PXF, their fellow eyes and age matched healthy controls. They found that RNFL in patients with PXS were significantly thinner than controls in all quadrants except the nasal quadrant and no significant difference was found in fellow eyes compared with controls except the temporal quadrant.^14^ Moreover they found significantly lower RNFL thickness in the inferior quadrant in PXF eyes compared to non PXF eyes that was similar to our results.^14^ Ozmen et al also reported significantly thinner average and inferior quadrant RNFL in PXF group as compared to control group.^1^

Most of the work done on measurement of RNFL thickness in PXF syndrome without glaucoma using different OCT machines revealed thinner mean global peripapillary RNFL in eyes with PXF as compared to control group with some variations in quadrantic measurements that may be attributed to variation in age, sample size, ethnicity and type of OCT machine used.^2^,^3^,^6^,^12^ Ozge et al in their study compared RNFL thickness in eyes with PXF glaucoma, PXF syndrome and healthy controls.^15^ They found out that RNFL thickness in all quadrants and average thickness was significantly low in PXF glaucoma eyes as compared to other groups, but RNFL thickness comparison between PXF syndrome and healthy control eyes did not show a significant difference except in infero temporal quadrant.^15^ Another interesting study by Rao et al evaluated clinical findings and peripapillary RNFL thickness in patients with unilateral and bilateral PXF syndrome and found out that bilateral cases with PXF were older (p < 0.01) and had thinner RNFL (p = 0.04) than unilateral cases.^16^

Although, results of our study corresponds with the results of international studies, some variation in absolute values was found that may be attributed to variation in age, ethnicity, gender distribution, machine used and normative data. Reported mean RNFL thickness of normal Pakistani population in various local studies was 94.46 µm, 99.02 µm and 102.38 µm using Topcon 3D Mark II-1000, Spectralis Heidelberg and Stratus OCT respectively.^7^,^10^,^17^ RNFL thickness values in our study were found to be lower than these values both in control and PXF group. Our study gives some insight into diagnosing pre perimetric glaucoma using OCT as PXF patients with normal IOP and visual fields were tested and found to be having thin RNFL than age matched adults.

Our study was conducted at a tertiary care hospital; hence the results cannot be implemented on general population. Furthermore as glaucoma is a progressive disease resulting in progressive loss of nerve tissue with optic nerve head and visual field changes, testing at a single point in time cannot be conclusive. More population based prospective studies in our setup evaluating the changes over time need to be conducted.

## CONCLUSION

PXF patients with normal IOP and visual fields have thin RNFL as compared to healthy age matched controls. Therefore routine assessment and follow up of PXF patients with OCT may help in early diagnosis of PXF glaucoma.

## References

[1] Ozmen MC, Aktas Z, Yildiz BK, Hasanreisoglu M, Hasanreisoglu B (2015). Retinal vessel diameters and their correlation with retinal nerve fiber layer thickness in patients with pseudoexfoliation syndrome. Int J Ophthalmol.

[2] Mohamed MM (2009). Detection of early glaucomatous damage in pseudo exfoliation syndrome by assessment of retinal nerve fiber layer thickness. Middle East Afr J Ophthalmol.

[3] Sorkhabi R, Rahbani MB, Ahoor MH, Manoochehri V (2012). Retinal nerve fiber layer and central corneal thickness in patients with exfoliation syndrome. Iran J Ophthalmol.

[4] Junejo SA, Jatoi SM, Khan NA, Qureshi MA (2008). To determine prevalence of Pseudo exfoliation at a Tertiary Eye Care Centre: A Hospital based study. Pak J Med Sci.

[5] Rao RQ, Arain TM, Ahad MA (2006). The prevalence of pseudoexfoliation syndrome in Pakistan. Hospital based study. BMC Ophthalmology.

[6] Naik RR, Bhalke VB (2015). Analysis of retinal nerve fiber layer thickness in patients with pseudoexfoliation syndrome using spectral domain optical coherence tomography. Int J Sci Res.

[7] Gondal TM, Qazi ZA, Jamil AZ, Jamil MH (2011). Accuracy of the retinal nerve fiber layer measurements by stratus optical coherence tomography for perimetric glaucoma. J Coll Physicians Surg Pak.

[8] Pan Y, Varma R (2011). Natural history of glaucoma. Indian J Ophthalmol.

[9] Mansoori T, Viswanath K, Balakrishna N (2011). Ability of spectral domain optical coherence tomography peripapillary retinal nerve fiber layer thickness measurements to identify early glaucoma. Indian J Ophthalmol.

[10] Fatima S, Islam QU, Ishaq M, Bajwa MS, Afridi JA (2015). Comparison of retinal nerve fiber layer thickness in patients of primary open angle glaucoma and healthy adults. Pak Armed Forces Med J.

[11] Mitchell P, Wang JJ, Hourihan F (1999). The relationship between glaucoma and pseudoexfoliation: the Blue Mountains Eye Study. Arch Ophthalmol.

[12] Eltutar K, Acar F, Ozturker ZK, Unsal E, Erkul SO (2015). Structural changes in pseudoexfoliation syndrome evaluated with spectral domain Optical coherence tomography. Curr Eye Res.

[13] Lisboa R, Leite MT, Zangwill LM, Tafreshi A, Weinreb RN, Medeiros FA (2012). Diagnosing pre perimetric glaucoma with spectral domain optical coherence tomography. Ophthalmology.

[14] Yüksel N, Altıntaş Ö, Çelik M, Özkan B, Çağlar Y (2007). Analysis of retinal nerve fiber layer thickness in patients with pseudo exfoliation syndrome using optical coherence tomography. Ophthalmologica.

[15] Ozge G, Koylu MT, Mumcuoglu T, Gundogan FC, Ozgonul C, Ayyildiz O (2016). Evaluation of retinal nerve fiber layer thickness and choroidal thickness in pseudoexfoliative glaucoma and pseudoexfoliation syndrome. Postgrad Med.

[16] Rao A (2012). Clinical and Optical Coherence Tomography Features in Unilateral versus Bilateral Pseudoexfoliation Syndrome. Ophthalmic Vis Res.

[17] Mukhtar S, Hassan N, Dawood Z, Zehra N (2015). Retinal Nerve Fiber Layer Thickness in a Subset of Karachi (Pakistan) Population. Br J Med Med Res.

